# Posterior, Extended, Pedicled Latissimus Dorsi Flap for the Reconstruction of a Large Complicated Lower Back Wound

**Published:** 2016-08-03

**Authors:** Michael Mirmanesh, Dan Mazzaferro, Zachary Borab, Lee L. Q. Pu

**Affiliations:** ^a^Division of Plastic Surgery, University of California Davis, Sacramento; ^b^Drexel University College of Medicine, Philadelphia, Pa

**Keywords:** posterior extended latissimus dorsi flap, complex lower back wound, exposed spinal hardware, latissimus dorsi flap, posterior trunk wound

## DESCRIPTION

A 53-year-old man with metastatic lung cancer who underwent urgent decompression and stabilization of a pathologic L4 burst fracture developed a chronic wound after surgical site breakdown following chemotherapy and radiation. The wound was 9 × 7 cm with exposed hardware at the base. A posterior, extended, pedicled latissimus dorsi myocutaneous flap was used to adequately close the wound.

## QUESTIONS

1. **Describe the reconstructive options for large posterior trunk wounds?**
2. **What are the key characteristics of a latissimus dorsi flap?**
3. **What is modified in a posterior extended latissimus dorsi flap?**
4. **Why use a pedicled flap instead of a turnover flap or free flap?**

## DISCUSSION

A variety of flaps are used depending on the size of the lower back defect. A more superficial, 2-dimensional defect may benefit from a local flap, but deep and complex 3-dimensional defects need additional tissue to fill and support the wound while also attempting to minimize compromising muscle function. Local muscle flaps are often a first-line treatment if there is adequate tissue; commonly used local muscle flaps include the reverse turnover latissimus dorsi flap and the gluteus maximus flap.^1^ Free flaps are considered when insufficient local tissue exists or when there is substantial damage to the surrounding soft tissue.^2^ A latissimus dorsi free flap is the most common reported free flap for extensive lower back coverage. Other free flaps described for lumbar defects include the transverse rectus abdominis, trapezius, and gluteus maximus.^3^

The latissimus dorsi flap can be harvested as a muscle or myocutaneous flap. It is classified as a Mathes and Nahai type V flap, as it has both a dominant pedicle (thoracodorsal) and secondary segmental (thoracic and lumbar perforators) blood supply. The pedicle length can reach 15 cm and is usually accompanied by a single venae comitantes. Flaps can be harvested up to 20 × 35 cm. The long pedicle and large size make this a workhorse flap in reconstruction.^4^

The posterior extended latissimus dorsi flap is a modification that serves to elongate the skin island inferior and medially. Dissection to include the muscle and subcutaneous tissue is performed with careful attention to the path of the descending branch of the thoracodorsal artery. This is vital to the survival of the flap. The insertion of the latissimus dorsi to the humerus is divided to provide more freedom to the pedicle. The modifications provide the flap with greater bulk and length at the risk of increased donor site morbidity, prolonged drainage, and seroma formation.^5^

In this patient, the size of the surgical resection and radiation damage substantiated the need for a free flap latissimus dorsi repair. It was originally intended to treat his large complex wound with a free latissimus dorsi flap anastomosed to the superior gluteal vessels. However, after adequate dissection and exposure, the superior gluteal vessels, particularly the vein, appeared to be small and not suitable for microvascular anastomosis. A latissimus turnover was considered, however; the segmental perforators were considered to be in the zone of radiation injury. A posterior extended latissimus dorsi myocutaneous flap was then utilized and inset into the defect. Had appropriate coverage not been possible with the posterior extended latissimus dorsi flap, we would have considered vein grafting to the superior gluteal arteries; however, they were initially deemed inadequate. In addition, the potential for complications with doubling the amount of microvascular anastomosis made this a less attractive option.

## Figures and Tables

**Figure 1:**
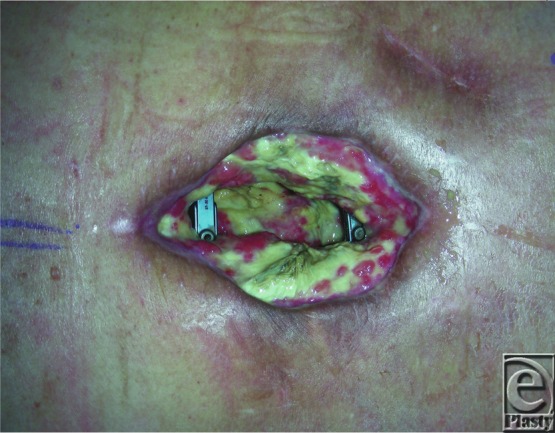
Chronic wound with exposed hardware and radiation tissue damage.

**Figure 2:**
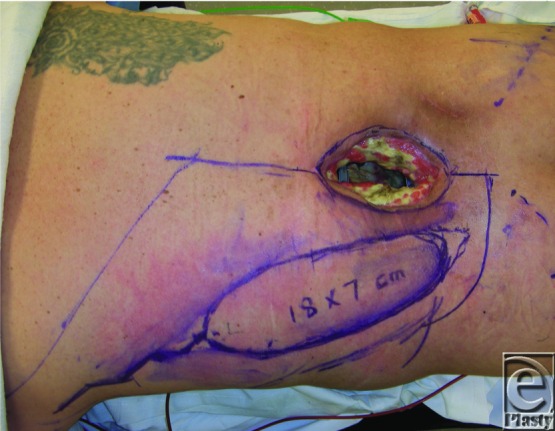
Skin paddle design.

**Figure 3:**
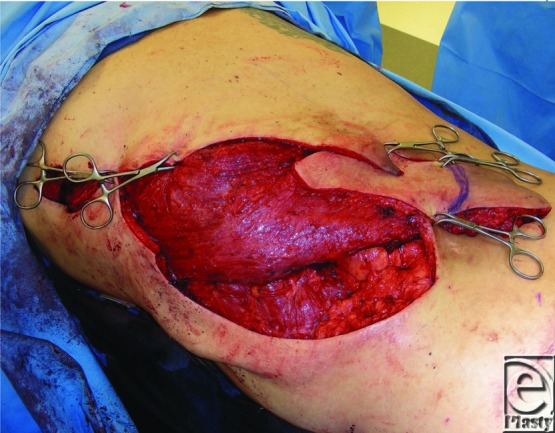
Flap and split-thickness skin graft prior to negative pressure dressing application.

**Figure 4:**
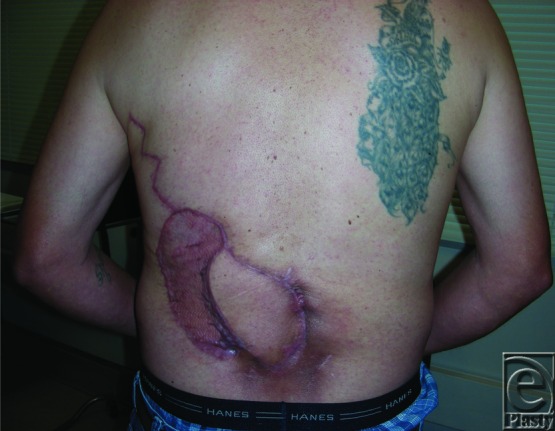
Well-healed reconstruction at 6 months postoperatively.
